# Effect of High-Fidelity Simulation on Medical Students’ Knowledge about Advanced Life Support: A Randomized Study

**DOI:** 10.1371/journal.pone.0125685

**Published:** 2015-05-08

**Authors:** Andrea Cortegiani, Vincenzo Russotto, Francesca Montalto, Pasquale Iozzo, Cesira Palmeri, Santi Maurizio Raineri, Antonino Giarratano

**Affiliations:** Department of Biopathology, Medical and Forensic Biotechnologies (DIBIMEF), Section of Anesthesiology, Analgesia, Intensive Care and Emergency, Policlinico “P. Giaccone”, University of Palermo, Palermo, Italy; Erasmus Medical Centre, NETHERLANDS

## Abstract

High-fidelity simulation (HFS) is a learning method which has proven effective in medical education for technical and non-technical skills. However, its effectiveness for knowledge acquisition is less validated. We performed a randomized study with the primary aim of investigating whether HFS, in association with frontal lessons, would improve knowledge about advanced life support (ALS), in comparison to frontal lessons only among medical students. The secondary aims were to evaluate the effect of HFS on knowledge acquisition of different sections of ALS and personal knowledge perception. Participants answered a pre-test questionnaire consisting of a subjective (evaluating personal perception of knowledge) and an objective section (measuring level of knowledge) containing 100 questions about algorithms, technical skills, team working/early warning scores/communication strategies according to ALS guidelines. All students participated in 3 frontal lessons before being randomized in group S, undergoing a HFS session, and group C, receiving no further interventions. After 10 days from the end of each intervention, both groups answered a questionnaire (post-test) with the same subjective section but a different objective one. The overall number of correct answers of the post-test was significantly higher in group S (mean 74.1, SD 11.2) than in group C (mean 65.5, SD 14.3), p = 0.0017, 95% C.I. 3.34 – 13.9. A significantly higher number of correct answers was reported in group S than in group C for questions investigating knowledge of algorithms (p = 0.0001; 95% C.I 2.22–5.99) and team working/early warning scores/communication strategies (p = 0.0060; 95% C.I 1.13–6.53). Students in group S showed a significantly higher score in the post-test subjective section (p = 0.0074). A lower proportion of students in group S confirmed their perception of knowledge compared to group C (p = 0.0079). HFS showed a beneficial effect on knowledge of ALS among medical students, especially for notions of algorithms and team working/early warning scores/communication.

## Introduction

High-fidelity simulation (HFS) is a learning method consisting on reproduction of medical scenarios through the use of a computerized manikin that can be programmed to recreate clinical conditions and to react to learners’ actions in a controlled and safe setting. [1–3] HFS has been largely investigated in emergency medicine and cardiopulmonary resuscitation [4–6] and it has been recognized as a pivotal learning method for technical and non-technical skills. [6–9] Advanced life support (ALS) is a set of life-saving protocols, skills and information aiming to support vital functions in cardiorespiratory arrest victims and other life-threatening situations developed by the European Resuscitation Council (ERC). [10] In this context, lack of knowledge is associated with mismanagement and poor patients’ outcome. [11] According to the theory of adult experiential learning by Kolb, the learning process consists of four circular phases: 1) concrete experience 2) reflective observation 3) abstract conceptualization 4) active experimentation. *Concrete experience* is the source for observation and reflection. During the second phase of *reflective observation*, the learner is involved in a process of attentive consideration of events, leading to creation of a general sense from the offered particular experience. The learner is then prone to actively test the product of the experiential learning in different situations, starting the circle again. [12] HFS provides the learners a realistic experience, the possibility to reflect on actions and to create abstract concepts, which may eventually be applied in new situations of their daily clinical practice. Through this process, knowledge may be created and/or reinforced by the transformation of experience. [3,12] Few studies have been designed with the primary aim of investigating the usefulness of HFS for knowledge acquisition in the setting of ALS and no studies investigated its effect on different categories of notions. [4,6] Moreover, the outcome of knowledge was not always assessed by a tool with enough sensitivity and specificity, possibly explaining the mild to moderate effect size of simulation, when compared to alternative learning methods, in previously published meta-analyses. [13–15] HFS is also costly and time-consuming, as it needs specialized personnel, equipment and spaces. [16] Therefore, its effectiveness for knowledge acquisition should be demonstrated by a study designed to assess the additional contribution of HFS for the learning process of ALS. The primary aim of our study was to compare frontal lessons, followed by a HFS session, with frontal lessons alone as a learning method for ALS among medical students. The secondary aims were to evaluate the effect of HFS on knowledge acquisition of different sections of ALS and personal knowledge perception of students.

## Methods

**Fig 1:**
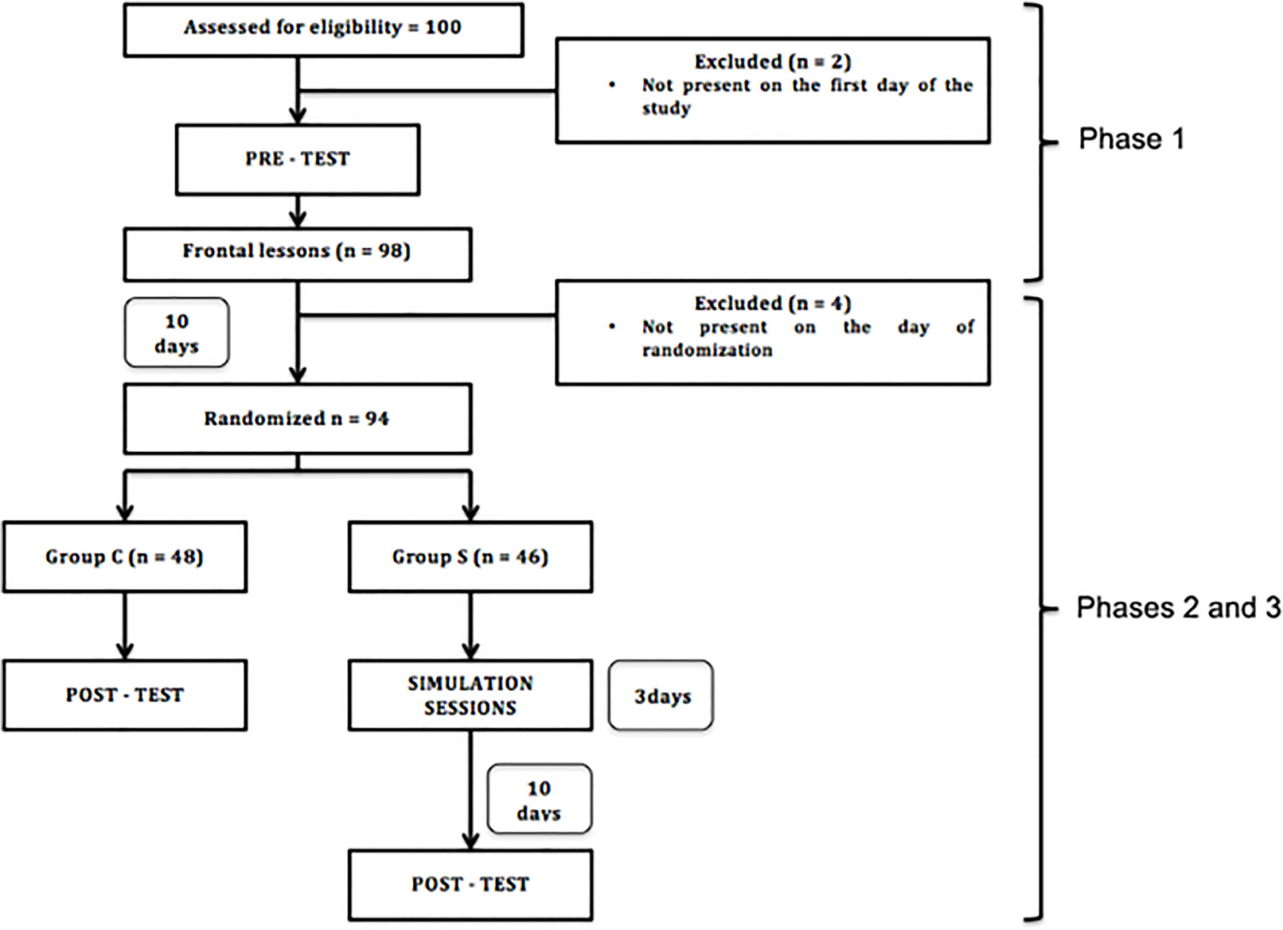
Flow-chart of the study.

We obtained the approval from the Ethics Committee of the University Hospital “P. Giaccone” (Palermo, Italy) for this single-centre, parallel-group, randomized study (allocation ratio 1:1). The study was performed from May to July 2014 at the simulation centre “Salvatore Mangione” of the University of Palermo. Our study consisted of three phases: 1) recruitment of participants, pre-test questionnaire, standard frontal lessons about ALS; 2) randomization in two groups: participation in a high-fidelity simulation session (groups S) versus no additional intervention (group C); 3) post-test questionnaire for both groups (Fig 1).

### Phase 1

We recruited participants through an announcement published in our institutional website. Students attending the last 3 years of medical school course were able to register. The link of registration was published 4 weeks before the beginning of the study and was available until the limit of 100 participants. An alphabetical list of participants was generated from the website and it was available on the first day of the study and their presence verified. Students were asked to give a written informed consent to participate in the study and to carefully read the information sheet explaining how to complete the questionnaires properly and the importance of filling in them by their own, without any external contribution. Each participant was assigned to an identification code. The list of participants with their respective identification codes was concealed to researchers involved in performing lessons, simulations and in the final evaluation of questionnaires. The pre–test consisted of two sections: 1) *Subjective section*: 9 questions investigating the personal perception of knowledge about ALS (with a Likert 1–10 scale rating system) 2) *Objective section*: 100 true-false questions about ALS (32 *Algorithms* questions: investigating knowledge about the sequence of actions to be performed according to ERC ALS guidelines [10] in case of adult cardiorespiratory arrest and other emergencies; 36 *Technical skills* questions: investigating the technical skills involved in ALS as chest compressions, defibrillation or monitoring; 32 *Team working/early warning scores/communication strategies* questions: investigating knowledge about score systems used for the early activation of medical emergency team (early warning scores), team working, team leading and communication strategies described in ALS guidelines. [10] Table 1 reports examples of questions from both sections. A maximum time of 2 hours was allowed to complete the questionnaire.

**Table 1:** Examples of questions from both the subjective and objective sections of the questionnaire.

*Objective section (true-false)*										
**ALGORITHMS**										
In case of non-shockable rhythms, epinephrine should be given as soon as IV access is achieved									True	
									False	
**TECHNICAL SKILLS**										
In a patient in cardiac arrest with a endotracheal tube in place, chest compressionsshould be alternated with ventilations in a 30:2 ratio									True	
									False	
**TEAM WORKING/EARLY WARNING SCORE/COMMUNICATION**										
The acronym SBAR stands for a communication strategy among health care Professionals									True	
									False	
*Subjective section (1-totally disagree; 10- totally agree)*										
I think to know the ALS algorithm for non shockable-rhythm										
1	2	3	4	5	6	7	8	9		10
I think to know all the principles of good communication among members of a Resuscitation team										
1	2	3	4	5	6	7	8	9		10

After completion of the pre-test, all participants attended 3 frontal lessons (each lasting 2 hours) on ALS. Lessons were taken by an academic researcher from the Department of Anesthesia, Intensive Care end Emergency Medicine, University of Palermo. All the lessons were taken according to the *set-dialogue-closure* scheme. [17] A syllabus describing in detail the topics to cover was previously written and a second researcher was present during lessons in order to verify that all topics were covered. A checklist was completed for this purpose.

### Phase 2—Phase 3

After 10 days from the last lesson, all participants were asked to come back to the simulation centre. Students randomized in the control group (Group C) were asked to answer the post-test questionnaire consisting of both *subjective* and *objective* sections. The *subjective* section was identical to the pre-test apart from one additional question asking whether the participant had changed his/her personal perception of knowledge in light of the training on ALS. The *objective* section consisted on 100 true-false questions, different from those included in the pre-test but still regarding the topics included in the syllabus. The same 2-hour interval of pre-test was allowed for its completion. Participants randomized in the group S were divided in 3 groups in order to complete the simulation sessions within 3 days. The simulation sessions were run by an instructor with a crisis resource management official certification (Institute of patient safety, inPASS). The high-fidelity simulator SimMan 2 by Laerdal (Stavanger, Norway) was used for the simulation sessions. During HFS, algorithms, technical skills and team working/early warning scores/communication strategies were incorporated into each scenario. At the beginning of each session, the students had the possibility to interact with the simulator, devices (e.g. defibrillators, monitors, airway management equipment) and environment. A student was randomly assigned as team leader and the remaining participants as team members. Each student participated in 3 different scenarios acting as team leader at least in one of them. An investigator completed a checklist of topics to be included in each scenario. Simulations sessions were conducted using the *on the fly* modality *with trends and handlers* in which the instructor maintains a full manual control of all simulator features and parameters with the addition of some pre-programmed sets of actions and physiological parameters modifications. [18] An instructor actively participated in the scenario as a healthcare provider not belonging to the ALS team with different duties according to the scenario.

At the end of each scenario, the instructor invited all participants of the day-session to join a debriefing, namely a learning conversation during which all participants were guided through a critical appraisal of their performance. We adopted the technique of *debriefing with good judgment* for this purpose. The instructor, during the debriefing, gave feedbacks to students adopting the technique of *advocacy with inquiry*. [17,19,20] Each debriefing session lasted from 2 to 3 times the duration of the corresponding scenario. [20] After 10 days from the last simulation session, all students randomized in Group S completed the post-test questionnaire. Post–test was identical to the one completed by Group C apart from an additional question investigating which intervention, among frontal lessons or HFS, had a major contribution to their opinion change about the previously given scores in the subjective section of the pre-test.

The primary outcome of the study was the overall number of correct answers of the post-test questionnaire between the two groups. Secondary outcomes were: 1) the post-test scores for each categories of knowledge, namely algorithms, technical skills and team working/early warning scores/communication 2) the Likert-scale score assessing personal perception of knowledge about ALS among the two groups. A post-hoc analysis was performed to assess which factors had an influence on primary outcome.

### Randomization

A researcher involved neither in frontal lessons nor in simulation sessions generated the random sequence using an on-line, random sequence generator. A simple randomization was performed. Randomization list was obtained after 10 days from the last lesson leading to allocation concealment of students during lessons and the following period (10 days) until the next phase of the study. The investigator involved in assessment of pre-test and post-test questionnaires was blinded to students’ name.

### Statistical analysis

We estimated that, with a sample size of 74, the study would have a 80% power to detect a minimum difference of 10 correct answers between the two groups (with α = 0.05). We expected a standard deviation (SD) of 15 in each group. The sample size was increased by 15% for non-parametric tests use (when appropriate). A further 15% was added for drop out/lost of follow-up students, according to our experience. [21] Therefore, we planned to include 50 students in each group. We analyzed variables distribution with the Kolmogorov-Smirnov test and checked the plot distribution of the data for detection of skewness. Homoscedasticity was tested by the F-test. We calculated and reported mean and SD for variables with a normal distribution. When homogeneity of variance was observed, a Student’s T-test was adopted for comparison of independent groups. In case of unequal variances, a Welch-test was performed and reported. We expressed variables without a normal distribution as median and interquartile ranges (IQR, 25^th^—75^th^) and comparisons were performed with Mann-Whitney U-test. Mann-Whitney U and the test statistic Z (corrected for ties) were reported as a measure for differences between groups. A frequency table was constructed and the chi-square test or Fisher’s exact test were used for comparisons of proportions. For the post-hoc analysis, a multiple regression model was constructed. An overall post-test score equal or higher than 70% in the objective section was adopted as dependent variable. The threshold of 70% of right answers in the post-test as a target of meaningful knowledge acquisition was selected by consensus before the study, according to previous experience. A stepwise method was used for inclusion of independent variables into the model. We tested the power of the calculated model by the Hosmer-Lemeshow goodness of fit test. We calculated and reported odds ratios as a measure of correlation between variables. Internal consistency of the subjective questionnaire was investigated by the Cronbach’s alpha statistic. [22] A two-tailed p < 0.05 was considered statistically significant. Statistical analysis was performed using MedCalc for Windows, version 9.5.0.0 (MedCalc Software, Mariakerke, Belgium). We adopted the Consolidated Standards of Reporting Trials (CONSORT) guidelines for this trial. [23] CONSORT checklist of the study is available as S1 CONSORT checklist.

## Results

One hundred students completed the on-line registration for study participation and were considered eligible. Two students were not present on the first day of the study and were excluded. Four students were not present on the day of randomization. A total of 94 students were then randomized (Fig 1). The randomization led to allocation of 46 students in Group S and 48 students in Group C. Table 2 shows the baseline characteristics and pre-test scores for the two groups.

**Table 2:** Demographic data and baseline scores of correct answers in pre—test questionnaire.

Demographic data and PRE—TEST scores		Group S		Group C	Overall		P—value
		(n = 46)		(n = 48)	(n = 94)		
**Age**		26.0		26.0	26.0		P = 0.72
(median, IQR)		(25.0–29.0)		(24.0–27.5)	(25.0–28.0)		
**Sex**		M = 43.5%		M = 52.1%	M = 47.9%		P = 0.42
		F = 56.5%		F = 47.9%	F = 52.1%		
**Year of Medical school**		4° = 36.9%		4° = 33.3	4° = 35.1%		P = 0.90
		5° = 26.1%		5° = 31.2%	5° = 28.7%		
		6° = 36.9%		6° = 35.4%	6° = 36.2%		
**Subjective section score** (median, IQR)		3.5		3.0	3.0		P = 0.48
		(1.0–6.0)		(1.0–6.0)	(1.0–6.0)		
**Objective section—Overall score of correct answers** (mean, SD)		33.8		30.2	32.0		P = 0.34
		(18.6)		(18.9)	(18.7)		
**Objective section ALGORITHMS** (mean, SD)	**Correct answers**	10.6		10.0	10.3		P = 0.67
		(6.0)		(7.2)	(6.6)		
	**Not-correct answers**	21.4		21.7	21.5		P = 0.85
		(6.01)		(7.17)	(6.59)		
**Objective Section TECHNICAL SKILLS** (mean, SD)	**Correct answers**	12.9		10.8	11.8		P = 0.18
		(7.65)		(6.99)	(7.35)		
	**Not-correct answers**	23.1		25.3	24.2		P = 0.16
		(7.68)		(7.15)	(7.46)		
**Objective Section COMMUNICATION** (mean, SD)	**Correct answers**	10.3		9.29	9.81		P = 0.50
		(7.3)		(7.77)	(7.52)		
	**Not-correct answers**	21.7	22.7			22.2	P = 0.50
		(7.29)	(7.79)			(7.52)	

The number of correct answers, in both the subjective and objective sections of the pre-test questionnaire, was not significantly different in the two groups. The overall number of correct answers of the post-test questionnaire was significantly higher in group S (mean 74.1, SD 11.2) than in group C (mean 65.5, SD 14.3), p = 0.0017, 95% C.I. 3.34–13.9. A significantly higher number of correct answers were reported in group S than in group C for questions investigating the knowledge of algorithms and communication strategies, whereas it was not possible to reject the null hypothesis of equal scores in both groups for the category of technical skills (Table 3).

**Table 3:** Number of correct and not-correct answers in post—test questionnaire.

POST—TEST scores		Group S		Group C		Overall		P—value
		(n = 46)		(n = 48)		(n = 94)		(95%CI of difference S-C)
**Subjective section score** (median, IQR)		8.0		6.5		7.0		P = 0.0074
		(7.0–9.0)		(6.0–8.0)		(6.0–8.0)		
**Objective section—Overall score of correct answers** (mean, SD)		74.1		65.5		69.7		P = 0.0017
		(11.2)		(14.3)		(13.5)		(3.34 to 13.9)
**Objective section—ALGORITHMS** (mean, SD)	**Correct answers**	26.0		21.9		25.5		P < 0.0001^2^
		(3.61)		(5.43)		(20.0–28.0)^1^		(2.22 to 5.99)
	**Not-correct answers**	5.96		10.1		6.5		P < 0.0001^2^
		(3.61)		(5.43)		(4.0–12.0)^1^		(-5.99 to -2.22)
**Objective Section TECHNICAL SKILLS** (mean, SD)	**Correct answers**	25.4		25.0		25.2		P = 0.67
		(4.78)		(5.11)		(4.93)		(-1.59 to 2.46)
	**Not-correct answers**	10.4		11.0		10.7		P = 0.61
		(4.86)		(5.11)		(4.97)		(-2.57 to 1.52)
**Objective Section COMMUNICATION** (mean, SD)	**Correct answers**	22.4		18.5		20.4		P = 0.0060
		(6.58)		(6.59)		(6.83)		(1.13 to 6.53)
	**Not-correct answers**	9.24	12.5		10.9		P = 0.0085	
		(5.64)	(6.26)		(6.16)		(-5.75 to—0.86)	

^1^ Median and IQR (Overall population not normally distributed).

^2^Welch-test.

**Fig 2:**
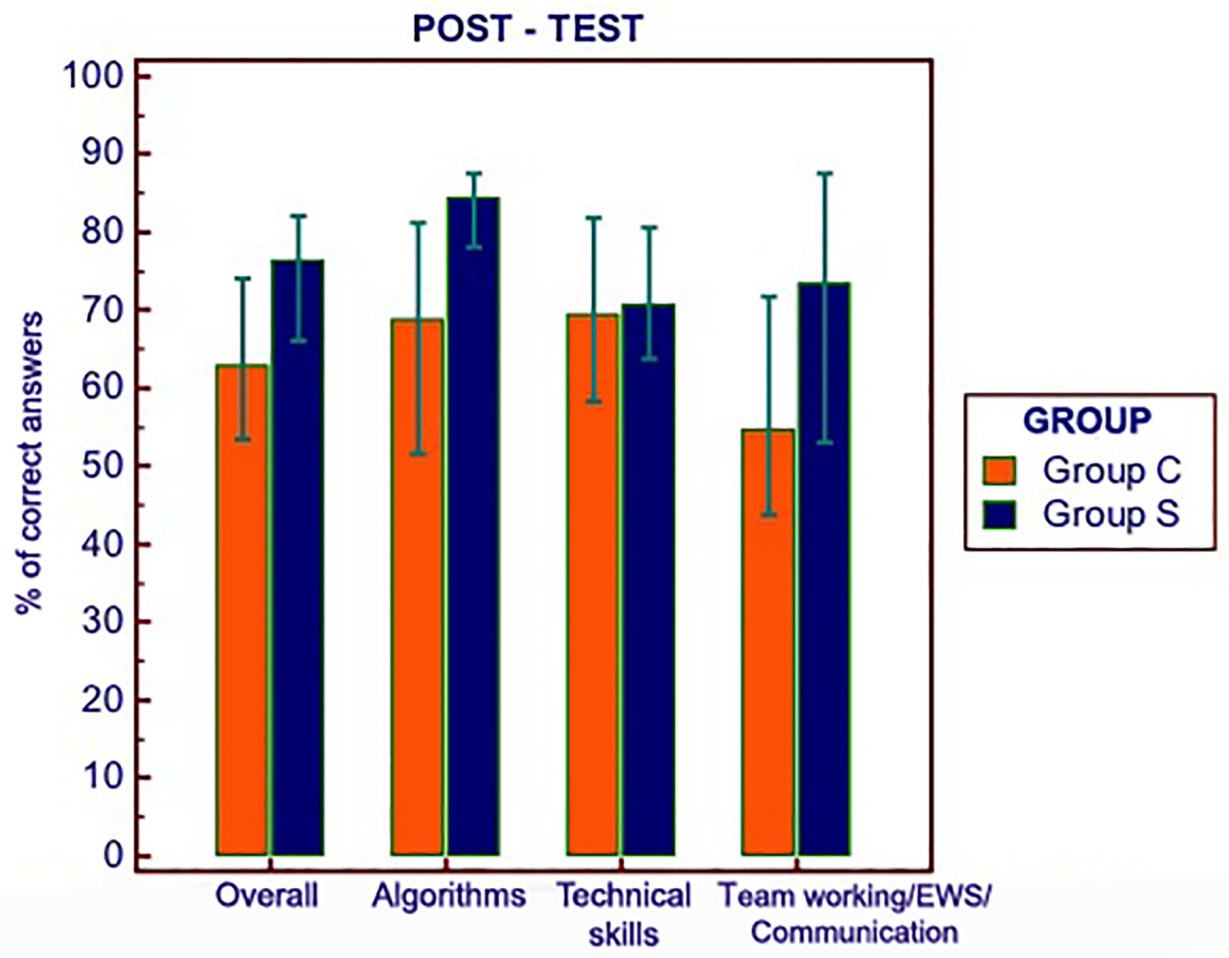
Post-test results in the objective section (% of correct answers and IQR). A p <0.05 was observed for overall, algorithms and team working/early warning scores/communication results (see Table 3); EWS: early warning scores.

Similar results were observed comparing the two groups according to the number of not correct answers (i.e. sum of wrong and not given answers). Students in group S showed a significantly higher overall score in the post-test subjective section (8.0, IQR 7.0–9.0) than students in group C (6.5, IQR 6.0–8.0), p = 0.0074, U = 756.50, Z = 2.68. The proportion of students confirming their previously given perception of knowledge in ALS was significantly different among the two groups (p = 0.0079), with 60.4% of subjects in group C (31/48) confirming their previously reported perception compared to 32.6% in group S (15/46). Among subjects in group S not confirming their perception, 74.2% (23/31) attributed their opinion change to HFS. Fig 2 shows a bar-chart with scores (expressed as percentage of correct answers) of the post-test objective questionnaire.

A significantly higher proportion of students in group S (33/46 = 71.7%), compared to group C (18/48 = 37.5%), reported a score ≥ 70% of overall correct answers (p = 0.001). A logistic regression was constructed using the post-test score equal or higher than 70% as dependent variable. A stepwise method was used for model construction. Group (either C or S), attending the last year of medical school and overall number of correct answers in the objective section of the pre-test were considered as independent variables. Both *group* and *pre-test score* resulted as independently associated with the selected outcome (group, OR 4.45, C.I. 1,74–11,3; pre-test score, OR 1.04, C.I. 1,02 to 1,07), with a Hosmer and Lemeshow goodness-of-fit test for the model of p = 0.72.

## Discussion

In our study, medical students participating to HFS sessions, in association with frontal lessons, showed a higher overall post-test score and a higher personal knowledge perception of ALS compared to those participating to frontal lessons only. Our study design aimed to test HFS as an additional learning tool for medical student and not as an alternative classic teaching method. The rationale behind this design is that participants to HFS session should have a certain grade of knowledge about the topic of HFS sessions in order to take the best out of all simulation phases. Background knowledge can be provided by preliminary teaching lessons, self-learning and/or previous clinical experience. [3] Since medical students in our study could have had heterogeneous background knowledge about ALS, we decided to standardize preliminary knowledge giving them the same information through frontal lessons. However, pre-test results showed no significant differences in baseline knowledge between the two groups.

Considering the different categories of knowledge, a significant difference in correct questions between the two groups was observed only for algorithms and team working/early warning scores/communication strategies. Consistent results were reported after considering not correct answers. Of note, we decided to analyze also not correct answers since we believe that knowledge acquisition may be measured either as a gain in correct answers in the post-test or a lesser number of not correct answers (wrong and not given answers). It may be argued that HFS enhances those notions requiring an interaction among team members or rapid adoption of sequence of actions. The advantage of HFS for learning notions about ALS algorithms may be explained by the reflective practice during each scenario and debriefing session. [3,9,19] Therefore, most students in group S referred to feel confident and able to recognize all possible rhythms of presentation of a cardiac arrest. However, during debriefing, they freely reported that living a HFS and experiencing the need for a rapid decision making in a stressful environment is different and this experience led them to reconsider their previously reported opinion about their knowledge of ALS. Team working is a key issue for safe and effective patient care. [24–26] Even if principles of leadership and team working has been established and embodied in curricula, it seems that HFS provides a useful contribution for their reinforcement. [27–29] Categories of team communication, leadership, coordination and decision-making have been included in a team performance framework influencing patient outcome. [30] These finding are particularly true for ALS and have been confirmed in previously published studies on simulation and further investigated in light of the underlying psychological dynamics. [30–32] We may argue that being involved in a HFS scenario as a team leader or team member had a positive influence in deep comprehension of these notions through their direct experience. Poor communication among team members has been recognized as a potential source of error and adverse events in critical care setting. [33,34] Communication strategies have been developed in order to provide healthcare practitioners a method for an effective transmission of information in a structured and organized manner. [35,36] In all scenarios of our study, we created events forcing participants to transmit information and orders to other colleagues through the adoption of a structured communication strategy. [10] We believe that this had a positive effect on knowledge acquisition about team working/communication among students in group S. This finding has been previously reported in studies investigating the benefit of simulation for team working during inter-professional scenarios. [37]

Participation in a HFS simulation session was independently associated with a higher post-test score since students who underwent a HFS showed a four times probability of scoring 70% or higher on post-test questionnaire. The pre-test score had a negligible, though significant, influence on a higher post-test scoring. We decided to include the variable *last year of medical school* in the logistic regression model since, in Italy, students attend lessons of ALS during the emergency medicine course during the last year. Another reason is that they may have more clinical experience than younger colleagues. However, attending the last year of medical school did not show a correlation with post-test score.

This study has limitations. Firstly, one may argue that part of the observed benefit of HFS on knowledge could be attributed simply to the additional learning intervention by itself, leading to a repetition of the same information, irrespective of its modality. However, we believe it is unlikely that any learning intervention would have provided the same effect on knowledge instead of HFS. The reason for this belief is that HFS is intrinsically different from any other intervention since the learner goes through the adult learning circle with his/her background without any passive acquisition/recalling of notions. Of note, according to our results, only specific categories of knowledge (algorithms and team working/early warning scores/communication) received a significant benefit from HFS.

It may be argued that our *2-versus-1* interventional design presents intrinsic limitations that could have been overcome adding another session of frontal lesson for the students who underwent only the first lesson. This strategy would have the value of comparing two educational strategy consisting of two interventions each (frontal lesson plus frontal lesson versus frontal lesson plus HFS) perhaps leading theoretically to a more balanced comparison. However, our study design was aimed to be pragmatic and reflecting the real world of medical education. Frontal lessons and HFS are nowadays the most common educational methods used in medicine and the aim of our design was to compare them in terms of efficacy in knowledge recreating the real educational practice where they are often associated. In our opinion, an association of two frontal lessons about the same topic within a short period of time would not reflect real educational practice and may lead to a difficult interpretation of the results and difficult application in daily activity by the educators.

Another limitation may be attributed to the lack of previous assessment of the sensitivity and specificity of our questionnaires for detection of the effect of the intervention. However, we decided to formulate 100 different questions for pre-test and post-test, exploring the categories of algorithms, technical skills and team working/early warning scores/communication in order to enhance the sensitivity and specificity of our tool. Another limitation is the difficult exact reproducibility of each scenario (depending on participants’ competence and interactions). However, we tried to standardize each scenario by including all knowledge categories described in the syllabus and further confirming their observation during simulation by a checklist. Before each scenario, we randomly selected a team leader and team members. Of note, all participants in each simulation scenario were invited to join a learning conversation with the instructor and other participants during the debriefing session. We preferred to administer the post-test questionnaire on a single day for all students in group S in order to avoid the possible diffusion of questions among participants performing the test on different days. Another limitation could be the additional learning activities of the students during the post-intervention period before the post-test. It is possible that, during this time period, students could have reviewed the information provided during lessons. We believe that it is not possible to avoid this event and we decided to provide the official ERC ALS guidelines [10] website link in order to standardize the source of information. Finally, another limitation is the single assessment of knowledge during the period following the interventions. However, we selected an intermediate time frame of 10 days from the end of both frontal lessons and HFS sessions, in order to avoid the previously reported *boosting effect* occurring soon after an educational intervention while excluding the possible influence of other, unpredictable factors (e.g. academic lessons, daily-work activities) which would have intervened in case of a longer follow-up. [17,20,38] Given the proven benefit of HFS, associated with frontal lessons, for knowledge of ALS among medical students when compared to standard frontal lessons only, future trials should avoid comparison of HFS with standard frontal lessons but, rather, should focus on which innovative instructional design of HFS may provide the best benefit for learning.

## Conclusion

HFS showed a beneficial effect on knowledge acquisition of ALS notions in medical students. Algorithms and team working/early warning scores/communication were the categories which resulted significantly influenced by HFS compared with frontal lessons alone. Moreover personal knowledge perception of knowledge was influenced by HFS.

## Supporting Information

S1 Consort Checklist (DOC)

S1 Dataset Dataset of the study. (XLSX)
